# Manuel D’Hydrosudopathie, Ou Traitement Des Maladies Par L’Eau Froide, La Sueur, L’Exercise, Et Le Régime; Suivant La Méthode Employée Par V. Priessnitz à Graeffenberg

**Published:** 1840-08-15

**Authors:** 


### BIBLIOGRAPHICAL NOTICE.

MANUEL D'HYDROSUDOPATHIE, *ou Traitement des Maladies par l'Eau Froide, la Sueur, l'Exercise, et le Régime; suivant la Méthode employée par V. Priessnitz à Graeffenberg.* Par le Dr. BIGEL. Paris: 1840.

*Manual of Hydrosudopathy, or the Treatment of Diseases by Cold Water, Sweating, Exercise, and Regimen; according to the method employed by V. Priessnitz at Graeffenberg.* By Dr. BIGEL.

WITHIN a few years past a novel method of treating disease has attained considerable notoriety in Germany, and may be regarded as the successor of Homœopathy. It is the treatment of all diseases by cold water. It matters little what the disease may be, the same curative method is applicable to it, with some little variation in the mode of its administration, but still the essential agent is the same,—that is, pure and cold water.

In acute diseases, cold water has been long used as a powerful adjuvant to other methods of treatment; rarely, however, has it been resorted to without the agency of some more powerful and more regularly admitted pharmaceutical agent: the new method is, therefore, little more than an extension of what was previously known. But in chronic diseases the case is different; water is not often resorted to, except in the form of mineral water, or of some ptisans which contain but a small proportion of medicinal substances, compared with the whole bulk of the remedies:—in these cases the active part of the remedy is of course the water. By the new method no medicinal substance whatever is used: the treatment is conducted simply by cold water, used both internally and externally, and profuse sweating. The sweats are not produced by warm drinks, or by any artificial heat, but by ablution with cold water, and afterwards by immersion in a cold plunging bath.

Many local affections are treated by compresses wrung out of cold water, or by the spout-bath; while the same agent is used in a different way as a revulsive,—that is, by immersing the feet in the water, and covering the vessel closely, so that the water becomes gradually heated, and acts as a warm pediluvium.

A method of treatment of this nature is necessarily most powerful, and is attended with too much trouble and positive suffering to be largely resorted to after the novelty of its introduction is passed. Thus far it has met with much favour; it is a most powerful alterative, and has probably been used with more discretion than will be practicable if it should be in troduced into general practice. That the treatment must either do much good or harm is obvious: the thorough saturation of the system, with the copious draughts of water which are forced out at every pore by the profuse sweating, renews, as it were, the whole body, and gives a new impetus to the function of nutrition. Hence, in many chronic, and even acute diseases, which are scarcely to be reached by ordinary medication, we may find the results equally unexpected and gratifying.

In our practice, we are in the habit of using cold water, both internally and externally, to a much greater extent than any of our professional brethren whose practice we have seen; and although we have never ventured to recommend a plan of treatment which even approaches the severe regimen of the hydrosudopathy, we are not surprised at its results; for we have often succeeded in curing chronic diseases which had exhausted the materia medica, by the free use of cold water. It is true we have rarely combined it with sweating; and we have not, as a general rule, given it internally with as much freedom as we could desire, as the majority of patients submit much more readily to affusions and baths, than consent to drench themselves with the abundant draughts necessary to produce the full alterative effects of the water. The boldness of the peasant Priessnitz may lead to a more extended use of the simple remedy which nature supplies with so much liberality, while a correct examination of its powers and dangers may restrict it within the limits in which it is of undoubted utility, and free from mischievous consequences.

We make some extracts from a very lively description of Priessnitz's establishment at Graeffenberg:

"Immediately upon my arrival at Graeffenberg," says M. Gross in his account, "the dinner bell rang, and I repaired to the dining table, which is ninety feet in length. Upon entering I was struck with the number of persons assembled: an hundred and sixty-eight individuals sitting indiscriminately, without distinction of age or rank, at three parallel tables. Priessnitz presided at the top of the first table on the left, where he is always present at breakfast, dinner, and supper. It is here that he gives his public audiences; he is constantly appealed to by his guests, each of whom has something to tell or ask him—the entire conversation being held without reserve or embarrassment. It is astonishing to witness so gay and lively a scene amidst such a collection of the sick. We had served soup, beef, veal, mutton, pork, poultry, with salad, and, what particularly struck me, enormous cucumbers, cooked with salt, which are here the order of the day. No other vegetables but cabbage and sourcrout; fresh butter for desert. Every one at table drinks a great deal of fresh water; from twenty to thirty glasses a day are the ordinary amount. To all his patients Priessnitz recommends the copious use of water, as well to repair the loss of liquid occasioned by the abundant daily perspirations, as for its remedial virtues.

"The whole aim in this system of treatment, tending to rouse the economy to activity, and give the vix medicatrix naturæ sufficient energy to expel morbific matter, Priessnitz, far from desiring to enfeeble the system by depriving it of nourishment, by prescribing a rigid diet, (with the exception of spices and alcoholic liquids, which are forbidden,) makes no interference with his patients' mode of living, and allows them to eat as much as they desire, encouraging the use of solid, nutritious, and easily digested food.

"After dinner," says M. Gross, "I consulted Priessnitz for my own complaint, a chronic cold in the head. The same evening, by his advice, I took a preparatory bath of about 18° of Reaumur, after which I was well rubbed down, and took a walk. Supper followed, which, as well as breakfast, consists of cold milk, fresh butter, and biscuits. At four the next morning I rose after a sound sleep, and proceeded to undergo my first sweating. A large blanket was spread on the floor, on which I stretched myself naked with the chamber-pot between my legs. The assistant then proceeded to wrap me so firmly in the blanket, that I could with difficulty stir. Next, a large feather bed, and afterwards the bed quilt, were placed on top of me, with my cloak on top of the whole; and, to conclude, my head was thrust in the pillow, with only the eyes, nose, and mouth free. Being naturally of a dry temperament, I was left two hours in this position, before the perspiration began to appear, though it usually breaks out much sooner. As soon as my host perceived that I was sweating, he opened the window, and gave me from time to time cold water to drink. This is done to refresh the lungs with the fresh air, to revive the strength of the body, and to prevent excessive heat, and the debility consequent thereon. Moreover, the cold water drunk while the perspiration is flowing, increases the activity of the perspiratory function, although it arrests it when drunk before the sweat breaks out. After having sweated for two hours, Priessnitz was satisfied, and came to my rescue: the duration of the sweating period varies from half an hour to four hours, and is never prolonged till debility is experienced. When rid of the bed, enveloped in my blanket, reeking with perspiration, I rapidly, with very comfortable sensations, descended the staircase to the bath. After washing my face and hands, and throwing off the blanket, I plunged at first into a bath of the same temperature as that of the preceding evening, which, after being rubbed down, I exchanged for one of natural coolness, to return again to the other; then, again, after a good rubbing down, to the cold bath, and back; finally, after another rubbing, to the tepid, whence I went muffled up to my chamber, wiped, and dressed myself, and afterwards exercised in the open air. I felt a delightful glow, and a particular elasticity of mind and body. The same process, not so long, was repeated in the evening. The day after, I plunged at once into the cold bath, taking, however the precaution, never to be neglected, of previously washing hands, face, and breast. It will be asked, why this sudden passage from heat to cold is not only harmless but beneficial, when daily experience shows the danger of drinking or bathing in cold water when overheated. The difficulty is solved by attending to the distinction between *active* and *passive* sweating. The former, produced by violent exercise, heats the blood, increases its circulation, and thus produces cutaneous perspiration. In this state of irritation, and agitation of the system, it is undoubtedly dangerous to use cold drinks or baths. But in *passive* sweating, in which the body is in a state of repose, preceding no heat or agitation, it is not only harmless but healthful to drink and bathe in cold water. *Fxperto crede.*"

The mode of application of the douche, and other interesting particulars, we have no room to notice. We recommend a perusal of the book, and attention to the subject, which is worthy the notice of every enlightened physician.

